# Thermal Management of Film Capacitors Using Graphene/Thermoplastic Polyurethane Composite Films

**DOI:** 10.3390/ma19214491

**Published:** 2026-10-25

**Authors:** Pengfei Liu, Ruihan Guo, Dongyin Niu, Qinghua Miao

**Affiliations:** 1 School of Energy and Architectural Engineering, Harbin University of Commerce, Harbin 150028, China; lpf1305432@126.com (P.L.); ray990118@126.com (R.G.); niudongyin@163.com (D.N.); 2 Heilongjiang Provincial Key Laboratory of Fluid Engineering Equipment and Digital Intelligence Technology, Harbin 150028, China; 3 National Innovation Center for Green and Low-Carbon Building Technology, Beijing 100032, China

**Keywords:** capacitor, thermal management, thermally conductive film, graphene, thermoplastic polyurethane

## Abstract

Thermal management is important for improving the safety, reliability, and service life of film capacitors operating under high-power conditions. In this study, graphene/thermoplastic polyurethane (TPU) composite films were prepared for capacitor heat dissipation. Physically modified graphene (G1) and chemically modified graphene (G2) were incorporated into TPU at loadings of 0.03, 0.06, and 0.30 wt%. The structural characteristics and chemical states of the films were investigated using X-ray diffraction (XRD), Raman spectroscopy, Fourier transform infrared spectroscopy (FT-IR), and X-ray photoelectron spectroscopy (XPS). The thermal conductivity and thermal diffusivity of the composite films were evaluated, and a numerical model was used to investigate their effect on capacitor thermal behavior. The results showed that graphene incorporation preserved the major chemical structure of the TPU matrix while modifying its structural and surface chemical characteristics. The thermal response depended on both graphene type and loading. Among the tested samples, the 0.06 wt% G1/TPU film exhibited the highest measured thermal conductivity of 0.236 W/(m∙K). The thermal diffusivity showed a nonmonotonic dependence on graphene loading, with the maximum values obtained at 0.06 wt% graphene. Numerical results indicated that the thermally conductive film improved heat-flux uniformity and reduced the temperature gradient within the capacitor model. These findings demonstrate the potential of graphene/TPU composite films as flexible thermal-interface materials for capacitor thermal management.

## 1. Introduction

Film capacitors are widely used in power electronics, electric vehicles, renewable-energy systems, and energy-storage devices. During high-power operation, electrical losses generate heat inside the capacitor, which may cause excessive temperature rise, thermal stress, performance degradation, and reduced service life [1,2,3,4]. Therefore, improving the thermal management capability of capacitors is important for ensuring their safe and stable operation.

Polymeric materials are commonly used in electronic devices because of their low density, electrical insulation, flexibility, processability, and chemical stability. However, most polymers have a low intrinsic thermal conductivity, typically on the order of 0.2 W/(m∙K), which limits their heat-dissipation performance [5,6,7]. Incorporation of thermally conductive fillers is an effective approach for improving thermal transport in polymer matrices. Graphene-, boron nitride-, graphite-, and MXene-based fillers have been investigated for improving the thermal conductivity, electrical insulation, and heat-spreading capability of polymer composite films [8,9,10,11,12]. Flexible heat-spreader and thermally conductive films have also been developed for thermal-management applications [13,14].

Graphene is a carbon nanomaterial with high intrinsic thermal conductivity, low density, chemical stability, and excellent mechanical properties. It has been widely investigated as a filler for thermally conductive polymer composites [15,16,17,18]. Recent studies have further explored graphene-based composites containing multi-walled carbon nanotubes, boron nitride, and thermoplastic polyurethane for constructing continuous heat-transfer pathways and improving thermal-management performance [19,20,21,22,23]. Graphene-containing nanocomposites have also been applied in functional devices, including supercapacitors and gas sensors [24,25]. Other composite-film systems based on graphite/diamond structures and thermally conductive dielectric polymers have been reported for heat dissipation and electrical insulation [26,27]. Graphene–carbon nanotube films and functionalized polyurethane/graphene nanocomposites have also shown potential as thermal-interface materials [28,29]. The mechanical reinforcement and self-healing characteristics of graphene/TPU films have been investigated in related studies [30,31], while conductive TPU composites have also been considered for multifunctional sensing and energy devices [32].

Thermoplastic polyurethane (TPU) is a flexible polymer with good mechanical properties, chemical resistance, and film-forming capability. Graphene/TPU composite films may therefore provide both flexibility and improved thermal transport for thermal-interface applications.

In this work, G1/TPU and G2/TPU composite films with graphene loadings of 0.03, 0.06, and 0.30 wt% were prepared by solution blending and casting. The structural features, chemical states, elemental compositions, thermal conductivity, and thermal diffusivity of the films were examined. The capacitor heat-dissipation performance of the selected composite film was further evaluated using ANSYS 2021 R1 numerical simulation.

## 2. Materials and Methods

### 2.1. Materials

Physically modified graphene (G1) and chemically modified graphene (G2), both with a stated purity of at least 99.5%, were obtained from Sinopharm Chemical Reagent Co., Shanghai, China. Thermoplastic polyurethane was supplied by Zhongshan Boreas New Material Co., Zhongshan, China. N,N-dimethylformamide (DMF, at least 99.5%) was purchased from Shanghai Macklin Biochemical Co., Ltd., Shanghai, China.

### 2.2. Preparation of Graphene/TPU Composite Films

TPU was dissolved in DMF to prepare a 30 wt% TPU solution. For composite-film preparation, G1 or G2 was added to 50 mL of TPU/DMF solution at graphene loadings of 0.03, 0.06, and 0.30 wt%. The graphene was added in several portions, while external water cooling was used to control the solution temperature. The mixture was stirred for 15 min and dispersed using a nano-disperser for 1 h, with the temperature kept below 30 °C.

**Figure 1:**
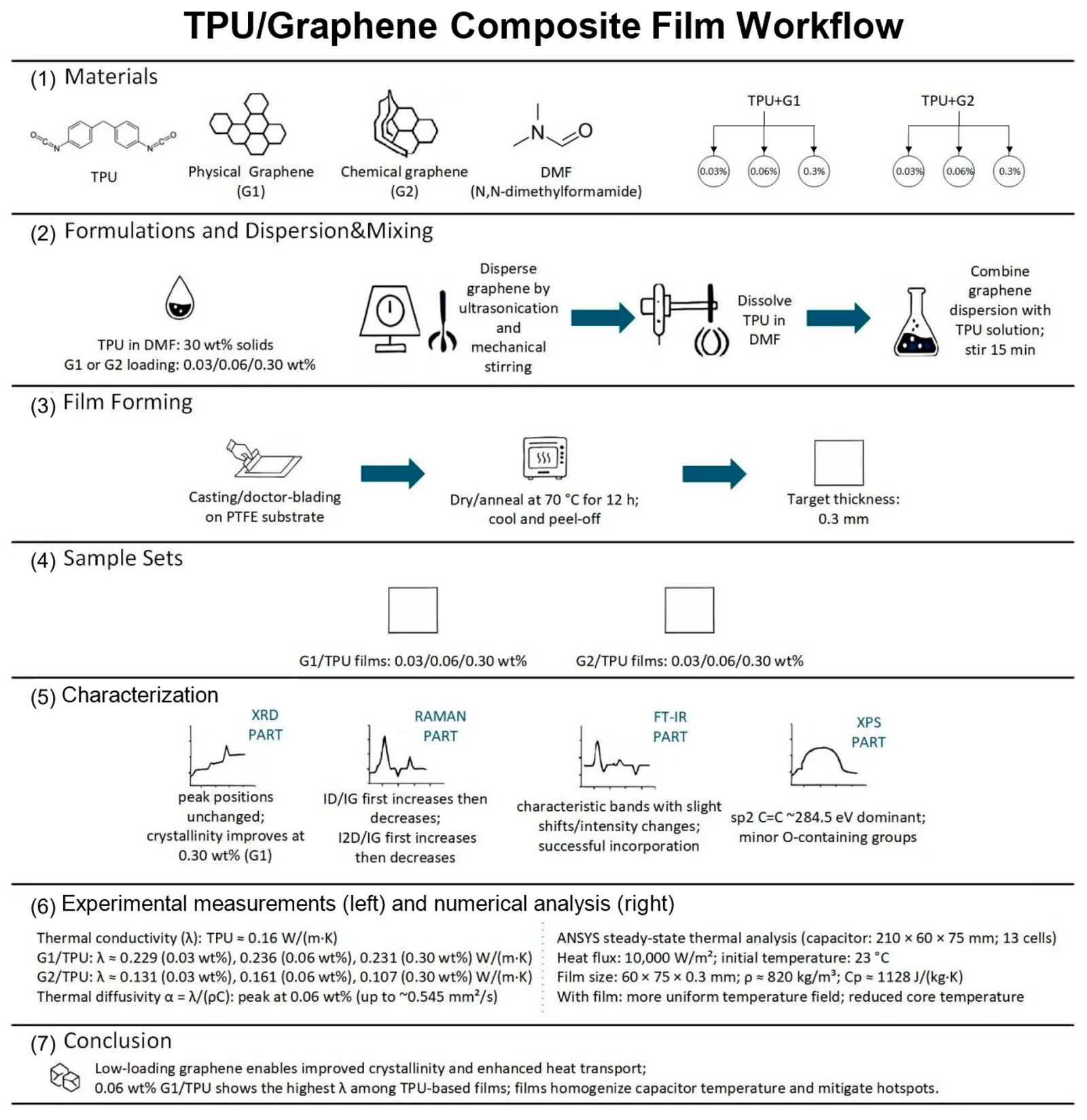
Schematic workflow for the preparation, characterization, and performance evaluation of graphene/TPU composite films.

The resulting suspension was cast onto a polytetrafluoroethylene (PTFE) substrate. The cast films were heated at 70 °C for 12 h until their mass became stable. After cooling, the films were peeled from the substrate. Neat TPU films were prepared using the same procedure without graphene. The preparation and evaluation workflow is shown in Figure 1.

### 2.3. Characterization and Thermal-Property Measurements

The crystal structure of the composite films was characterized via XRD. XRD measurements were performed using a MiniFlex 600 diffractometer (Rigaku, Akishima, Japan) with Cu Kα radiation (Kα1 wavelength 1.540593 Å), operated at 40 kV and 15 mA. Continuous θ–2θ scans were acquired from 5° to 90° with a 0.01° step and a scan speed of 10°/min, using a DeteX detector. Raman spectroscopy was used to analyze the graphitic structure and defect-related features of graphene. A LabRAM HR Evolution spectrometer (HORIBA France SAS, Palaiseau, France) was used with 532.04 nm excitation, a 100× objective, and a 600 grooves/mm grating. The recorded spectral range was approximately 50–3199 cm^−1^, with an acquisition time of 20 s and one accumulation per acquisition window. FT-IR spectroscopy was used to identify characteristic functional groups. Spectra were collected with a Nicolet iS20 spectrometer (Thermo Scientific, Madison, WI, USA) using a diamond attenuated total reflection (ATR) accessory, a DTGS detector, and a KBr beamsplitter. The range was 400–4000 cm^−1^ at 4 cm^−1^ resolution, with 32 sample scans and 32 background scans. XPS was used to analyze the elemental composition and carbon chemical states. The service order identifies the instrument as an ESCALAB 250Xi (Thermo Scientific, East Grinstead, UK). The acquisition files record an X-ray energy of 1486.68 eV, pass energies of 100 eV for survey spectra and 30 eV for high-resolution spectra, and energy steps of 1.0 and 0.1 eV, respectively.

The thermal conductivity of the films was measured using a TC3000E thermal conductivity analyzer (Xi’an Xiatech Electronic Technology Co., Ltd., Xi’an, China) using the hot-wire method at 35 °C. The sensor was placed between two specimens and held in position with a weight. Five measurements were averaged for each reported conductivity value. Thermal diffusivity was calculated according to Equation (1):

(1) $$\alpha =\frac{\lambda }{\rho \cdot C}$$

where *α*, thermal diffusion coefficient of the thermal conductive film, mm^2^/s; *λ*, the thermal conductivity of the film, W/(m∙K); *ρ*, density of the thermal film, kg/m^3^; *C*, specific heat capacity of the thermal conductive film, J/(kg∙K).

### 2.4. Numerical Model of the Capacitor

A three-dimensional capacitor model with dimensions of 210 mm × 60 mm × 75 mm was established in ANSYS 2021 R1. The model included 13 cells connected in series. Capacitor models with and without the thermally conductive film were constructed and meshed using a free-meshing method with local refinement.

The applied heat flux was 10,000 W/m^2^, and the initial temperature was set to 23 °C. The thermally conductive film had dimensions of 60 mm × 75 mm × 0.3 mm. The film with the highest measured thermal conductivity, 0.06 wt% G1/TPU, was selected for numerical analysis. The thermophysical parameters of the selected film are listed in Table 1.

## 3. Results and Discussion

### 3.1. Structural Characteristics of Graphene/TPU Composite Films

**Figure 2:**
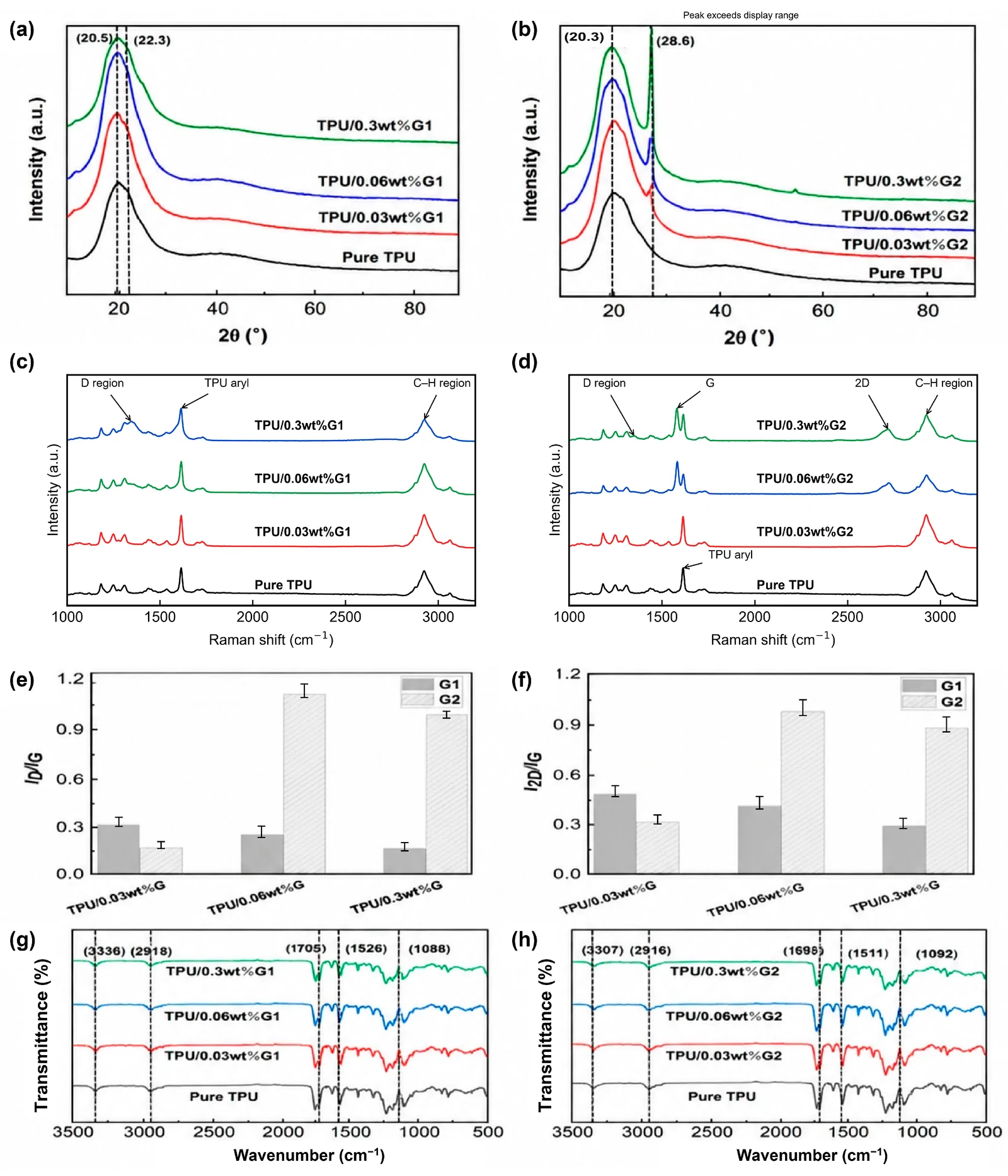
Structural characterization of graphene/TPU composite films: (**a**,**b**) XRD patterns of G1/TPU and G2/TPU films; (**c**,**d**) Raman spectra using the original manuscript labels; (**e**) ID/IG ratios; (**f**) I2D/IG ratios; and (**g**,**h**) FT-IR spectra of G1/TPU and G2/TPU films. In (**c**,**d**), spectra were smoothed with an 11-point, third-order Savitzky–Golay filter, independently normalized and vertically offset. Arrows locate measured maxima. D region and C–H region denote potentially overlapping bands; TPU aryl denotes the TPU aromatic-ring contribution. The bottom arrow in (**d**) targets the lowest trace. No separate G or 2D peak is assigned in (**c**). Author-review status: restored formulation labels remain unverified; panels (**e**,**f**) are unchanged, and their ratios have not been revalidated.

The XRD patterns, Raman spectra, ID/IG and I2D/IG intensity ratios, and FT-IR spectra are presented in Figure 2. The XRD patterns of the G1/TPU and G2/TPU films retained the broad diffraction features of TPU [33]. The diffraction peak positions changed only slightly after graphene addition, indicating that graphene incorporation did not substantially change the main crystalline structure of the TPU matrix.

For the G1/TPU films, the diffraction features became sharper at 0.30 wt% graphene. A similar trend was observed for the G2/TPU films. The characteristic graphene-related diffraction signal was observed near 26.6 degrees for G1 [33] and near 28.6 degrees for G2. These results indicate the presence of graphene within the TPU matrix [17].

The Raman spectra in Figure 2c,d are displayed from 1000 to 3199 cm^−1^. The D band is activated by defects or edges in sp^2^ carbon, whereas the G band arises from the in-plane vibration of sp^2^ carbon atoms. The 2D band is a two-phonon feature near 2700 cm^−1^ [34]. The numbered records contain a broad contribution near 1344 cm^−1^ in ID 4 and distinct features near 1581–1582 and 2720–2722 cm^−1^ in IDs 6 and 7, consistent with the D, G and 2D regions. TPU contributes bands near 1183 and 1249 cm^−1^ associated with ether/urethane and amide III vibrations, respectively, and near 1309 and 1439 cm^−1^ associated with coupled backbone and C–H deformation modes [35]. The bands near 1536, 1615, 1730 and 2924 cm^−1^ are assigned to urethane-related vibrations, aromatic-ring stretching, carbonyl stretching and aliphatic C–H stretching, respectively [36]. The feature previously labeled at 2898 cm^−1^ lies in the C–H stretching region, not the graphene 2D region. The D-region and C–H-region labels identify spectral regions rather than uniquely resolved components; overlapping polymer or carbon contributions are not separated here. The TPU aryl label identifies the aromatic-ring contribution near 1613–1615 cm^−1^, distinct from the G-region feature near 1580 cm^−1^; it does not imply an exclusive assignment of a composite band. The Raman analysis is therefore confined to the measured film spectra and literature-supported band assignments [34,35,36]; it does not establish the standalone Raman characteristics of G1 or G2.

Here, ID/IG and I2D/IG denote the D-to-G and 2D-to-G Raman intensity ratios, respectively. ID/IG characterizes defect-related disorder in carbon materials within the applicable disorder regime. I2D/IG is sensitive to graphene layer number and stacking and is interpreted together with the 2D-band shape and width [34]. Doping, excitation conditions and overlap with TPU vibrations also influence the measured ratios. Neither ratio directly measures the thermal conductivity of a composite; heat transport additionally depends on filler dispersion, interfacial thermal resistance and conductive pathways. Without standalone filler spectra and validated separation of the overlapping polymer and carbon bands, the ratios in Figure 2e,f are not used here to rank the intrinsic defect density or layer number of G1 and G2.

The FT-IR spectra retained the characteristic bands of TPU. The N–H stretching, carbonyl stretching, N–H bending, C–O stretching and C–O–C stretching bands reflect the principal functional groups of the matrix [37,38,39]. Inspection of the available numbered FT-IR records 1–7 resolves two overlapping carbonyl maxima at approximately 1699 and 1726 cm^−1^. Their sampled maxima span 1698.98–1699.46 and 1726.46–1726.94 cm^−1^, respectively. These differences are below 0.5 cm^−1^ at the recorded spectral resolution of 4 cm^−1^, so no resolved displacement is established for this set of records. The lower-frequency contribution is consistent with more strongly hydrogen-bonded carbonyl environments, while the higher-frequency contribution includes less strongly associated or free carbonyl groups; soft-segment chemistry also affects the component assignments [40]. The two contributions form an asymmetric carbonyl envelope with a high-wavenumber shoulder. Their relative intensities, rather than a resolved peak displacement, describe the observed profile differences.

### 3.2. XPS Analysis and Elemental Composition

**Figure 3:**
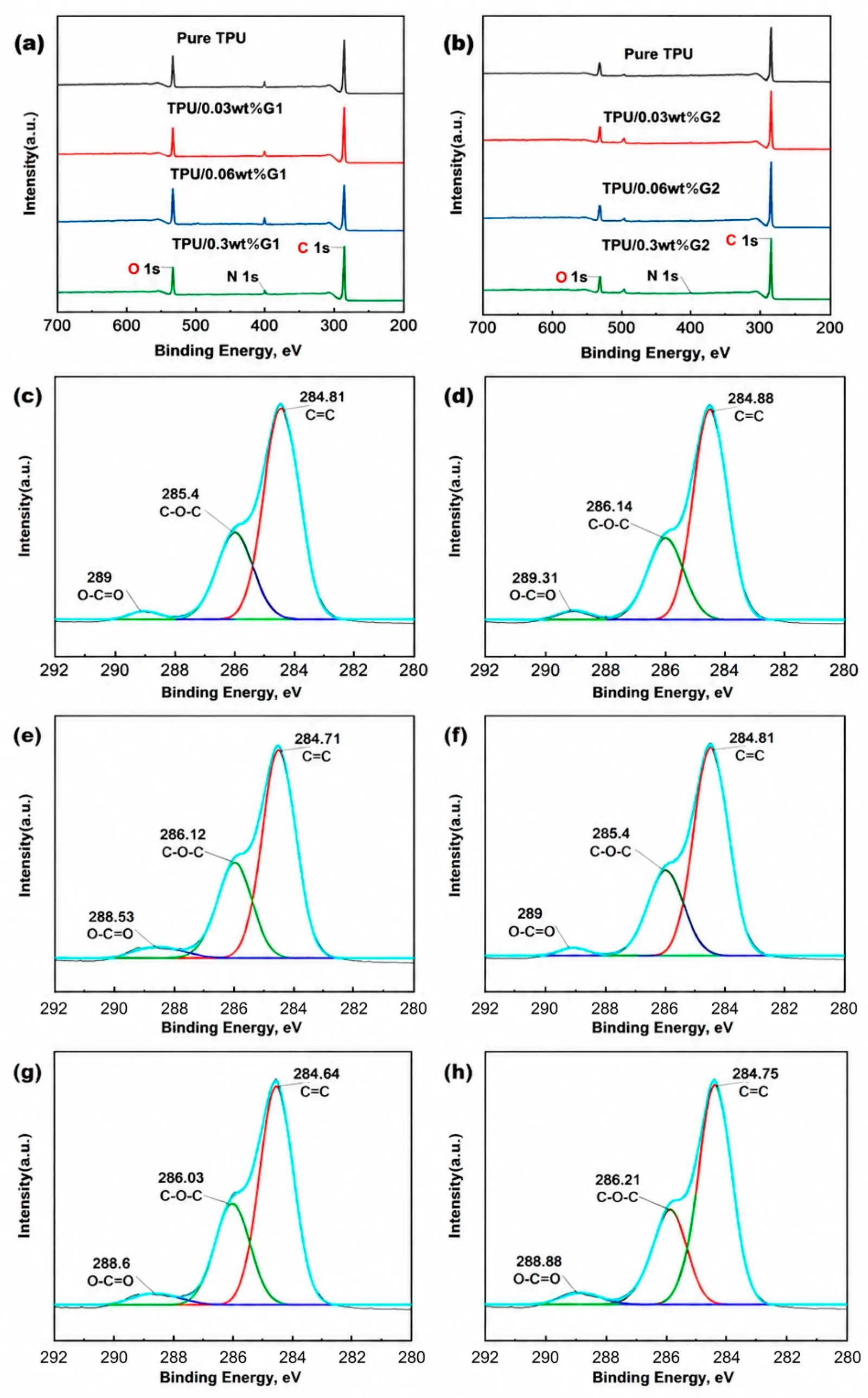
XPS analysis of graphene/TPU composite films: (**a**) XPS survey spectra of G1/TPU films; (**b**) XPS survey spectra of G2/TPU films; (**c**–**e**) C 1s fitted spectra of G1/TPU films containing 0.03, 0.06, and 0.30 wt% graphene, respectively; and (**f**–**h**) C 1s fitted spectra of G2/TPU films containing 0.03, 0.06, and 0.30 wt% graphene, respectively. In (**a**,**b**), the black, red, blue, and green lines represent neat TPU and composites containing 0.03, 0.06, and 0.30 wt% graphene, respectively. In (**c**–**h**), the cyan traces show the overall C 1s spectral profiles, and the red component peaks correspond to the C=C assignment indicated in the panels. In (**c**), the blue and green component peaks correspond to C–O–C and O–C=O, respectively. In (**d**–**h**), the green and blue component peaks correspond to C–O–C and O–C=O, respectively.

Figure 3 shows the XPS survey spectra and fitted C1s spectra of the composite films. The survey spectra confirmed the presence of C, N, and O in the graphene/TPU composite films. The main C 1s signal near 284.5 eV was associated with C–C or C = C bonding [41,42]. In the survey spectra, the C 1s and O 1s signals occur near 285 and 530 eV, respectively [41].

The fitted C 1s spectra showed components associated with carbon–carbon bonds, C–O–C groups, and oxygen-containing carbon groups [41]. The retention of these signals after graphene incorporation indicates that the principal chemical groups of TPU were maintained in the composite films [19,20,21]. The relative intensities of the fitted components varied among the samples, suggesting that graphene type and graphene loading affected the local chemical environment at the film surface.

The elemental compositions measured via XPS are listed in Table 2. For G1/TPU, the carbon content varied from 71.16 to 79.07 wt%, while the oxygen content varied from 14.62 to 19.70 wt%. For G2/TPU, the carbon content ranged from 70.91 to 74.99 wt%. These changes may be related to differences in graphene surface chemistry, dispersion state, and local surface heterogeneity.

Because XPS is a surface-sensitive technique, the measured elemental contents should not be interpreted as bulk composition values. The XPS results provide evidence of graphene incorporation and changes in surface chemical environments, but they do not alone establish the mechanism of thermal-conductivity enhancement.

### 3.3. Thermal Conductivity and Thermal Diffusivity

The thermal conductivity values of the composite films are listed in Table 3. Neat TPU exhibited a thermal conductivity of approximately 0.160 W/(m∙K). For G1/TPU, the thermal conductivity values were 0.229, 0.236, and 0.231 W/(m∙K) at graphene loadings of 0.03, 0.06, and 0.30 wt%, respectively. For G2/TPU, the values were 0.131, 0.161, and 0.107 W/(m∙K), respectively.

The highest thermal conductivity, 0.236 W/(m∙K), was obtained for the G1/TPU composite containing 0.06 wt% graphene. The results did not show a monotonic increase in thermal conductivity with graphene loading. Instead, an intermediate graphene loading produced the best thermal conductivity. This behavior may be associated with a balance between graphene dispersion, interfacial thermal resistance, and the formation of conductive pathways in the TPU matrix. Thus, 0.06 wt% G1/TPU is the optimal formulation among those tested in this study for thermal transport, exhibiting the highest thermal conductivity of 0.236 W/(m·K) and thermal diffusivity of 0.545 mm^2^/s.

**Figure 4:**
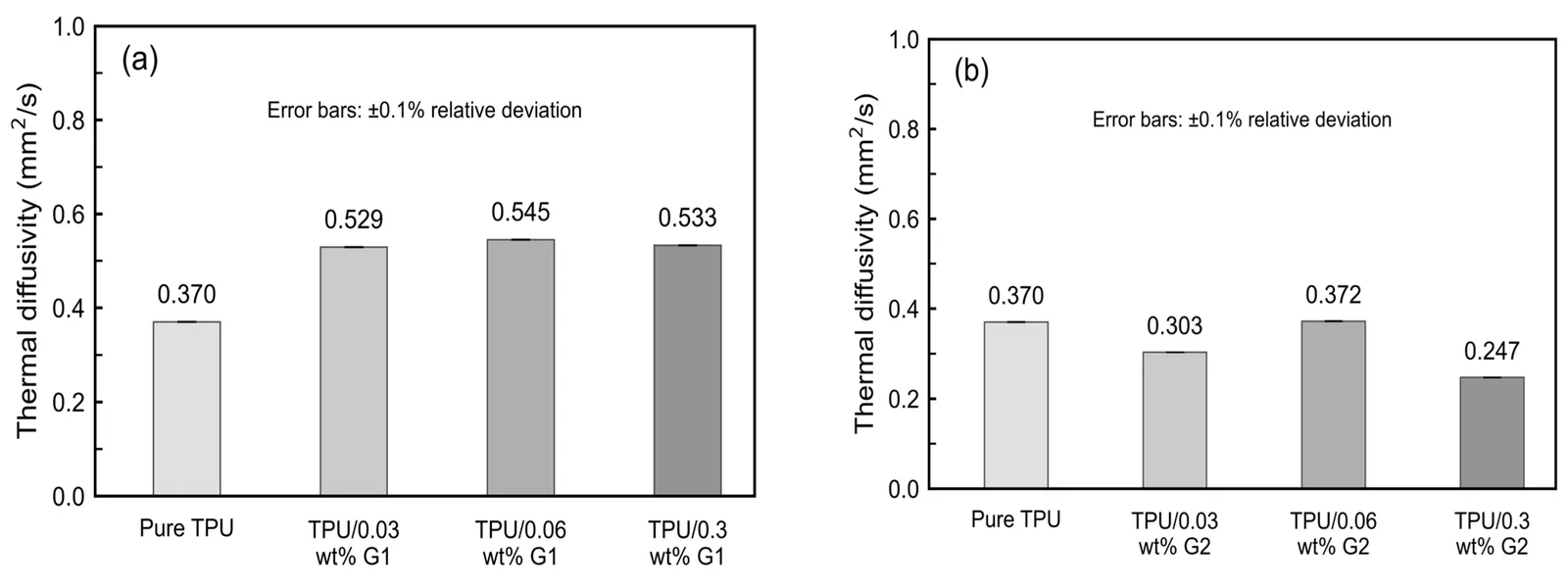
Thermal diffusivity of graphene/TPU composite films: (**a**) G1/TPU films containing physically modified graphene; and (**b**) G2/TPU films containing chemically modified graphene.

The thermal diffusivity results are shown in Figure 4. Both G1/TPU and G2/TPU films showed a decrease–increase–decrease trend with increasing graphene content. The highest thermal diffusivity values were observed at 0.06 wt% graphene. According to the original data, the maximum thermal diffusivity was 0.545 mm^2^/s for the physically modified graphene composite film and 0.372 mm^2^/s for the chemically modified graphene composite film.

### 3.4. Numerical Analysis of Capacitor Heat Dissipation

**Figure 5:**
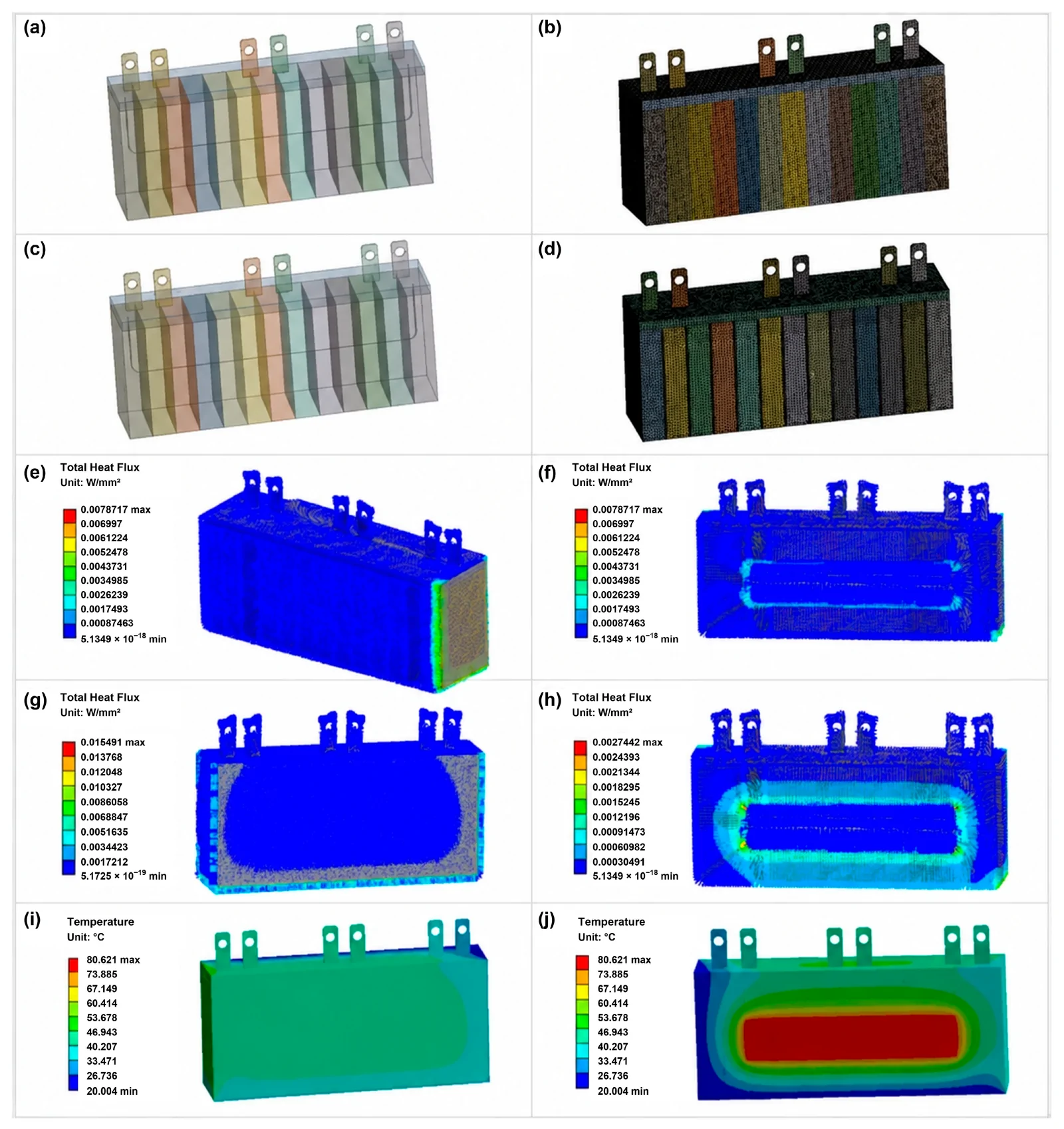
Numerical model and thermal-simulation results of the capacitor with and without the graphene/TPU composite film. (**a**) Geometric model of the capacitor without the thermally conductive film; (**b**) mesh model of the capacitor without the thermally conductive film; (**c**) geometric model of the capacitor with the thermally conductive film; (**d**) mesh model of the capacitor with the thermally conductive film; (**e**) external total heat-flux contour of the capacitor without the film; (**f**) internal or cross-sectional total heat-flux contour of the capacitor without the film; (**g**) external total heat-flux contour of the capacitor with the film; (**h**) internal or cross-sectional total heat-flux contour of the capacitor with the film; (**i**) external temperature contour of the capacitor with the film; and (**j**) internal or cross-sectional temperature contour of the capacitor with the film.

Figure 5 presents the capacitor geometry, mesh models, and numerical thermal results. Figure 5a–d compare the capacitor model and mesh with and without the thermally conductive film. Figure 5e,f show the simulation results for the capacitor without the film, whereas Figure 5g–j show the heat-flux and temperature distributions after application of the graphene/TPU composite film. The addition of the film improved the spatial distribution of heat transfer and reduced the temperature gradient between the internal region and the external surface.

Without the thermally conductive film, the temperature distribution was nonuniform, and the central region exhibited a higher temperature than the outer surface. The original simulation result indicated that the maximum internal temperature approached 80 °C under the selected boundary conditions.

After the graphene/TPU composite film was applied, the heat-flux distribution became more uniform. The temperature gradient between the internal region and outer surface was reduced, indicating improved heat-spreading performance. The composite film increased the effective heat-transfer area of the capacitor and promoted heat dissipation from the high-temperature region.

The measured thermal conductivity provides the link between material characterization and the numerical model. The selected 0.06 wt% G1/TPU film has a conductivity of 0.236 W/(m·K), compared with 0.160 W/(m·K) for neat TPU. Graphene incorporation is represented through the effective properties of the composite film rather than explicit graphene-sheet geometry. The numerical model evaluates how this film redistributes heat under the prescribed capacitor geometry and thermal loading, connecting the measured material property to the predicted heat-flux and temperature fields.

## 4. Conclusions

Graphene/TPU composite films containing G1 or G2 graphene at loadings of 0.03, 0.06, and 0.30 wt% were prepared and evaluated for capacitor thermal management. XRD, Raman, FT-IR, and XPS analyses showed that graphene incorporation modified the structural and surface chemical characteristics of the films while retaining the principal chemical structure of the TPU matrix.

The thermal properties depended on both graphene type and graphene loading. The highest thermal conductivity of 0.236 W/(m∙K) was obtained for the G1/TPU film containing 0.06 wt% graphene. The thermal diffusivity also reached its highest value at 0.06 wt% graphene. These results indicate that an intermediate graphene loading was more favorable than a higher loading under the present preparation conditions.

ANSYS simulation showed that the graphene/TPU thermally conductive film improved heat-flux uniformity and reduced the temperature gradient in the capacitor model. The composite film therefore has potential as a flexible thermal-interface material for improving capacitor heat dissipation. Further work should include repeated thermal-property measurements, uncertainty analysis, electrical-insulation measurements, morphology characterization, and experimental validation of the numerical model.

## Figures and Tables

**Table 1:** Composite thermally conductive thin-film parameters.

Density (kg/m^3^)	Specific Heat Capacity (J/kg∙K)	Thermal Conductivity (W/m∙K)
820	1128 J/(kg.K)	0.449/0.236

**Table 2:** Elemental composition of graphene/TPU composite films determined via XPS.

	C(wt%)	N(wt%)	O(wt%)
TPU	76.58	6.55	16.87
TPU/0.03 wt%G1	78.87	6.51	14.62
TPU/0.06 wt%G1	71.16	9.14	19.7
TPU/0.3 wt%G1	79.07	6.27	14.66
TPU/0.03 wt%G2	74.99	7.27	17.75
TPU/0.06 wt%G2	70.91	9.07	20.02
TPU/0.3 wt%G2	72.23	8.49	19.29

**Table 3:** Thermal conductivity of graphene/TPU composite films.

Graphene Addition Amount	TPU	G1	G2
0.03 wt%	0.16 W/(m∙K)	0.229 W/(m∙K)	0.131 W/(m∙K)
0.06 wt%		0.236 W/(m∙K)	0.161 W/(m∙K)
0.3 wt%		0.231 W/(m∙K)	0.107 W/(m∙K)

## Data Availability

The data that support the findings of this study are available from the corresponding author upon reasonable request.
